# Cost-Utility and Clinical Impact of a Multicomponent Maternal Safety and High-Dependency Care Model in Three Colombian Caribbean Hospitals: A Before-and-After Study

**DOI:** 10.5334/aogh.5403

**Published:** 2026-10-16

**Authors:** José Rojas-Suárez, Cintia Durán, Nestor Lora, Laura Briñez, Abril Calderón, Walter Annicchiarico-López, Nelson Alvis-Zakzuk, Larry Laza, Andrea Peña, Juan Carlos Correa, Mauricio Vasco-Ramirez, María Escobar-Vidarte, Javier Carvajal, Jairo Guerrero, Carmelo Dueñas-Castell, Rafael Navarro-España, José Santacruz-Arias

**Affiliations:** 1 Intensive Care and Obstetric Research Group (GRICIO), Department of Obstetrics and Gynaecology, Faculty of Medicine, Universidad de Cartagena, Cartagena, Colombia; 2 GINUMED Research Group, Corporación Universitaria Rafael Núñez, Cartagena, Colombia; 3 ESE Hospital San José de Maicao, Maicao, La Guajira, Colombia; 4 ALZAK Foundation, Cartagena de Indias, Bolívar, Colombia; 5 ESE Hospital La Divina Misericordia, Magangué, Bolívar, Colombia; 6 Clínica CEDES, Riohacha, La Guajira, Colombia; 7 Universidad CES, Medellin, Colombia; 8 INCINTA Consultores S.A.S., Cali, Colombia; 9 Secretaria Distrital de Salud de Cartagena, Bolívar, Colombia; 10 Postgrado de Medicina Crítica, Universidad del Sinú, Cartagena, Colombia

**Keywords:** maternal safety, high-dependency unit, severe maternal morbidity, cost-utility analysis, before-and-after study, middle-income country

## Abstract

*Background:* Obstetric high-dependency units may improve rescue care when embedded within broader maternal safety strategies.

*Objective:* To evaluate a multi-component maternal safety and technological strengthening model, including obstetric high-dependency capacity, across three Colombian Caribbean institutions with different governance models.

*Methods:* We conducted a multisite before-and-after implementation evaluation within a Ministry-funded maternal safety project. The model was implemented in one private clinic and two public hospitals in La Guajira and Bolívar, Colombia, with staggered implementation and varying data maturity. Data sources included billing records, administrative databases, and monthly quality indicators. Costs, severe maternal morbidity indicators, and longitudinal quality metrics were analysed by institutional implementation window. Pandemic-period variation from 2018 to 2023 was treated descriptively. Interrupted time series analyses were performed where post-implementation data allowed.

*Findings:* Implementation was associated with heterogeneous but generally favourable patterns. At Clínica CEDES, costs increased after implementation, while intensive care utilisation and several severe maternal morbidity indicators declined. At ESE Hospital San José de Maicao, mature post-implementation economic and conventional pre/post-clinical comparisons were unavailable; however, interrupted time series analyses showed favourable changes in longitudinal quality indicators, including reductions in weighted adverse outcome score (WAOS) level (*β* = −8.30, *P* = 0.0016) and slope (*β* = −1.20, *P* = 0.0064), adverse outcome index (AOI) level (*β* = −1.13, *P* = 0.0032), and severity index (SI) slope (*β* = −0.27, *P* = 0.030). ESE Hospital La Divina Misericordia showed mixed early clinical signals and descriptive quality improvement, but follow-up was limited.

*Conclusions:* A multi-component maternal safety model including obstetric high-dependency capacity may improve maternal care in high-risk settings, although effects vary by institutional context. High-dependency units appear most effective as part of an integrated rescue system rather than isolated infrastructure.

## Introduction

Maternal mortality (MM) and severe maternal morbidity (SMM) continue to represent major public health challenges in low- and middle-income countries. Although global strategies emphasise timely and high-quality obstetric care, preventable deaths and near-miss events persist in settings where health systems face limitations in the early detection of deterioration, escalation capacity, workforce preparedness, and institutional governance [1, 2]. These problems are particularly relevant in regions with high social vulnerability. In Colombia, in the department of La Guajira, the MM ratio reached 189.7 per 100,000 live births in 2019, approximately four times the national figure of 47.1 [3], whereas Bolívar reported the highest SMM ratio in the country in 2021, at 89.4 per 1000 live births [4]. More than 61% of maternal deaths in La Guajira between 2010 and 2012 occurred among indigenous women, reflecting deep ethnic and geographic disparities [5]. Geographic fragmentation, poverty, migration, and intercultural barriers further complicate access to appropriate care. To our knowledge, no published estimates of the economic cost of SMM or maternal safety interventions are available for our region. In this context, strengthening maternal rescue capacity requires more than intensive care beds. It requires an integrated continuum of recognition, infrastructure, response, and recovery, supported by human factors such as non-technical skills and inter-professional communication, which are critical to preventing adverse events [6].

Maternal mortality and maternal near-miss events constitute reference standards for maternal health surveillance promoted by the World Health Organization (WHO) and the Pan American Health Organization (PAHO) and adopted in Colombia through SIVIGILA [7, 8]. Although essential for monitoring and accountability, these measures have limitations as institutional quality indicators: maternal deaths are rare at the facility level, near-miss definitions vary across frameworks, and dichotomous counts do not capture severity or more frequent adverse obstetric outcomes [9]. Composite indicators such as the adverse outcome index (AOI), weighted adverse outcome score (WAOS), and severity index (SI) can therefore complement surveillance data by summarising event frequency, severity-weighted burden, and seriousness of adverse outcomes over time [10].

Obstetric high-dependency units (HDUs) have been proposed as a pragmatic solution to provide enhanced monitoring and intermediate care for women who are too unstable for standard wards but do not necessarily require intensive care [6, 11]. However, the available evidence indicates that the impact of these units is strongly influenced by the broader institutional system in which they operate. High-dependency beds implemented without trained teams, standardised protocols, reliable escalation pathways, and adequate support services have shown limited or inconsistent benefits [12]. In low- and middle-income settings, where resources are constrained and case complexity is high, isolated structural interventions are unlikely to produce sustained improvements unless they are embedded in multi-component strategies that simultaneously strengthen clinical processes, human resources, monitoring systems, and institutional learning.

Recent studies have demonstrated that multi-component maternal safety interventions, including care bundles, structured training, and continuous quality monitoring, can reduce severe morbidity and improve resource utilisation when implemented as integrated packages [13]. Economic evaluations of such interventions in resource-limited settings have suggested favourable cost-effectiveness profiles, particularly when they reduce intensive care admissions, hospital length of stay, and long-term disability. Nevertheless, most published evidence comes from single-centre studies or low-income countries, and there is limited information on how these models perform when scaled across institutions with different governance structures, operational models, and baseline capacities in middle-income countries [14].

The **SAFEX** (Safety and Excellence in Maternal Care) project was developed as a multi-component regional strategy to strengthen maternal and perinatal safety in high-priority areas of the Colombian Caribbean. The intervention combined situational diagnosis, workforce development, obstetric safety standards, technological and infrastructure support, high-dependency obstetric capacity, clinical escalation processes, adverse outcome monitoring, and health-economic evaluation. This study aimed to evaluate the implementation of this multi-component maternal safety and technological strengthening model, including obstetric high-dependency capacity, across three institutional settings with distinct organisational arrangements in the Colombian Caribbean, describing changes in costs, SMM indicators, and longitudinal quality outcomes.

## Methods

### Study design

We conducted a multisite implementation evaluation nested within a regional maternal and perinatal safety project funded by the Colombian Ministry of Science, Technology and Innovation (Minciencias). The parent project followed a quasi-experimental before-and-after design with staggered, non-randomised implementation across participating institutions. Given that implementation timing, data maturity, and operational context differed substantially across sites, we adopted a convergent multi-dimensional analytic approach rather than attempting a single pooled effect estimate. Economic, clinical, and longitudinal quality indicators were analysed and interpreted according to each institution’s implementation timeline and available data. The reporting of this study was guided by the STROBE statement for observational studies [15], the SQUIRE guidelines for quality improvement initiatives [16], and the Consolidated Health Economic Evaluation Reporting Standards 2022 (CHEERS 2022) for health economic evaluations, as applicable to the exploratory economic analyses [17].

### Setting

This project included three institutions in the Colombian Caribbean region with distinct governance and operational models: (1) Clínica CEDES, a private institution in Riohacha, La Guajira, providing medium- and high-complexity obstetric care; (2) Empresa Social del Estado (ESE) Hospital San José de Maicao, a public state-owned healthcare provider in Maicao, La Guajira, operating under national government intervention through the *Superintendencia Nacional de Salud*, Colombia’s national health regulatory and supervisory authority; and (3) ESE Hospital La Divina Misericordia, a public state-owned healthcare provider in Magangué, Bolívar, whose operation is supported by a private entity. We selected these institutions because they represent different organisational arrangements commonly found in high-priority maternal health regions in Colombia and because they serve populations with significant social and geographic vulnerability. Table 1 summarises the institutional models, implementation periods, and available data sources.

**Table 1:** Institutional models and data availability.

INSTITUTION	INSTITUTIONAL MODEL	LOCATION	IMPLEMENTATION PERIOD	MAIN AVAILABLE EVIDENCE
Clínica CEDES	Private institution	Riohacha, La Guajira	January 2022 onwards	Pre/post costs, exploratory cost-effectiveness, clinical outcomes, AOI/WAOS/SI
ESE Hospital San José de Maicao	Public ESE under national government intervention	Maicao, La Guajira	2024 onwards	Baseline economic data only for 2018–2023; post-implementation longitudinal AOI/WAOS/SI from 2024 onwards
ESE Hospital La Divina Misericordia	Public ESE with private operational support	Magangué, Bolívar	2024 onwards	Pre/post partial economic and clinical data, WAOS

### Population

The target population included obstetric patients of any gestational age and up to 42 days post-partum who received care at the participating institutions during each institution’s observation window. No exclusion criteria were applied in the institutional economic analyses because the objective was to evaluate real-world institutional service use and maternal care costs.

### Intervention

This study evaluated an intervention that was not limited to creating or operating an obstetric HDU. It consisted of a multi-component institutional model designed to improve maternal and perinatal safety in high-priority regions of the Colombian Caribbean. Table 2 summarises the components of the multicomponent model and their expected mechanisms.

**Table 2:** Components of the multi-component maternal safety and technological strengthening model.

COMPONENT	DESCRIPTION	EXPECTED MECHANISM
Situational diagnosis	Assessment of institutional gaps in infrastructure, workforce, technology, monitoring, escalation, governance, and leadership alignment	Identification of correctable system failures
Safety standards	Adaptation of clinical pathways for obstetric emergencies and severe maternal morbidity	Earlier recognition and standardised response
Obstetric high-dependency capacity	Intermediate care platform for high-risk obstetric patients	Enhanced monitoring and prevention of deterioration
Workforce strengthening	Training in technical competencies, Non-Technical Skills, team dynamics, and interprofessional communication	Improved preparedness and team response
Technological support	Equipment, monitoring capacity, oxygen support, laboratory readiness, and infrastructure	Better detection and treatment capability
Indicator monitoring	AOI, WAOS, SI, clinical outcomes, cost indicators, and dashboards	Continuous feedback and institutional learning
Economic evaluation	Cost description and exploratory cost-effectiveness analysis	Assessment of resource implications and sustainability

1. **Situational diagnosis and readiness assessment.** Identification of gaps in infrastructure, workforce, clinical processes, escalation, monitoring, data systems, governance, and leadership alignment.
2. **Obstetric safety standards and escalation pathways.** Adaptation of clinical pathways for SMM, obstetric emergencies, admission criteria, and escalation processes.
3. **High-dependency obstetric capacity and technological support.** Strengthening of monitoring, equipment, oxygen support, laboratory readiness, and physical spaces for enhanced obstetric care.
4. **Workforce development.** Training for clinical, administrative, and leadership teams, including technical competencies, inter-professional communication, and Non-Technical Skills through Colapso Materno® simulation-based training [18].
5. **Indicator monitoring and economic evaluation.** Development of dashboards, adverse outcome indicators, cost descriptions, and exploratory cost-effectiveness analyses to support feedback, learning, and sustainability assessment.

Following prior implementation evidence, the SAFEX components were delivered as an integrated package rather than as separable interventions [19]. This reflected the premise that high-dependency infrastructure requires trained teams, standardised pathways, reliable communication, leadership alignment, and continuous indicator feedback to improve rescue care. Conversely, training and protocols are unlikely to be effective without the monitoring capacity, equipment, and protected care environment needed for timely action. The model was therefore evaluated as a maternal rescue system, not as isolated infrastructure.

### Exposure definition

The primary goal was to implement a multi-component maternal safety and technological strengthening model. The implementation dates were defined according to the formal start of the structured intervention package at each site. In Clínica CEDES, the post-implementation period began in January 2022. In ESE Hospital La Divina Misericordia, structured implementation began in 2024. In ESE Hospital San José de Maicao, full model implementation occurred after the period covered by the available economic baseline data.

Because the COVID-19 pandemic introduced exceptional, non-systematic disruptions to maternal healthcare delivery, referral pathways, service use, resource allocation, and MM in approximately 2020, we did not use pandemic-period information to estimate intervention effects on clinical outcomes or longitudinal quality indicators. We treated any pandemic-related variation in service use or costs during the 2018–2023 economic observation period as a descriptive context, not evidence of intervention-related change.

### Data sources

Data were obtained from institutional billing records, RIPS (Registro Individual de Prestación de Servicios; national administrative health service records) datasets, administrative cost databases, SIVIGILA reports on SMM and mortality, internal project monitoring files, and monthly institutional quality indicator dashboards.

### Economic evaluation

Economic analyses were conducted from the perspective of the Colombian health system payer. Costs were expressed in Colombian pesos (COP). Institutional reports distinguished between direct accounting costs (billed services related to maternal events) and indirect or shared costs (human resources, equipment, infrastructure, and utilities). Where possible, a microcosting approach was used. A maternal event was defined using a 45-day window linking related episodes of maternal care. Total cost for each maternal event was calculated as the sum of directly billed costs recorded within the ≤45-day maternal episode and the allocated share of institutional personnel, equipment, infrastructure, and operating costs attributable to maternal care. For each calendar year, the annual mean total cost per maternal event was calculated by summing total costs across maternal events and dividing by the number of maternal events observed during that year. We allocated shared costs using institution-specific activity-based drivers based on resource use and the availability of administrative data. Exploratory cost-effectiveness analyses were performed at sites where sufficient pre- and post-implementation data were available. Incremental cost-effectiveness ratios (ICERs) were calculated as the difference in mean cost between periods divided by the difference in selected clinical outcomes (the number of hospital days avoided, intensive care stay avoided, SMM complications avoided, and maternal deaths avoided). These analyses were considered exploratory because of variation in post-implementation observation windows and limited adjustment for case severity.

### Clinical outcomes

Clinical outcomes included the number of maternal patients, maternal care events, hospital length of stay, intensive care length of stay, the proportion of maternal patients admitted to intensive care, the number of SMM cases and complications, SMM-related diseases, organ dysfunction, and MM. SMM cases were those notified by each institution to the Colombian national public health surveillance system (SIVIGILA) under the SMM (Morbilidad Materna Extrema) protocol, which classifies cases according to three groups of inclusion criteria: (i) disease-specific criteria (eclampsia, septic shock, and hypovolaemic shock); (ii) organ dysfunction criteria (cardiac, vascular, renal, hepatic, metabolic, respiratory, or coagulation dysfunction); and (iii) management-based criteria (admission to an intensive care unit, ICU; emergency surgical intervention; and transfusion of three or more units of blood products) [8]. SMM-related diseases referred to the underlying obstetric conditions recorded for each case, which included hypertensive disorders of pregnancy (severe preeclampsia, eclampsia, and haemolysis, elevated liver enzymes and low platelet count [HELLP] syndrome), obstetric haemorrhage, and pregnancy-related sepsis or severe infection, whereas organ dysfunction, intensive care admission, and maternal death were recorded as separate outcomes; these categories were not mutually exclusive, and a single patient could contribute to more than one. We applied the same case definitions and notification procedures before and after implementation. Because operational definitions of SMM differ across surveillance frameworks (e.g., the WHO near-miss organ-dysfunction criteria, ICD-based indicators used by the U.S. Centers for Disease Control and Prevention, and the broader Latin American criteria adopted by SIVIGILA), absolute frequencies are not directly comparable across systems [2]. These outcomes were therefore complemented with three longitudinal quality indicators (AOI, WAOS, and SI) capturing adverse outcomes.

### Longitudinal quality indicators

Three institutional obstetric quality indicators derived from the instrument developed by Mann et al. were used [10]: the AOI, the proportion of deliveries with at least one of the 10 prespecified adverse outcomes: maternal death, intrapartum or neonatal death, uterine rupture, maternal admission to an ICU, birth trauma, return to the operating room or delivery room, admission to a neonatal ICU, five-minute Apgar score <7, blood transfusion, and third- or fourth-degree perineal tear, interpreted as event frequency; the WAOS, the sum of severity-WAOS divided by the total number of deliveries, interpreted as the weighted institutional burden of adverse outcomes; and the SI, the sum of severity-weighted scores divided by the number of deliveries with at least one adverse outcome, interpreted as the clinical seriousness of events among affected patients. The severity weights followed those of the original instrument, and all the indicators were calculated monthly at each site. These indicators were interpreted as complementary signals of institutional obstetric safety rather than as definitive measures of preventability. Recent applications of AOI, WAOS, and SI have emphasised that such composite indicators can identify adverse outcome patterns from routine obstetric data but may not distinguish preventable events from unavoidable complications and may incompletely capture improvements driven primarily by teamwork, communication, safety culture, or escalation behaviours when these do not immediately translate into measurable clinical outcome reductions [20].

### Statistical analysis

Continuous variables are summarised as the means with standard deviations or medians with interquartile ranges, as appropriate. Categorical variables are summarised as counts and percentages.

Economic and clinical results were presented descriptively by institution. Monthly quality indicators were analysed using interrupted time series (ITS) methods, as previously proposed for studies similar to this [12, 21]. All analyses used available institutional datasets without patient-level linkages across institutions. When the post-implementation follow-up was sufficient (Clínica CEDES and ESE Hospital San José de Maicao), segmented regression models were fitted with a baseline trend term, an immediate level-change term, and a post-implementation slope-change term. WAOS and SI were modelled as continuous outcomes using linear segmented regression, and the AOI was modelled on the logit scale using a binomial/quasi-binomial generalised linear model with monthly deliveries as the denominator. Coefficients are reported with 95% confidence intervals and *P*-values. At ESE Hospital La Divina Misericordia, the short post-implementation window precluded model fitting, and the trends are presented descriptively, with a vertical line indicating the implementation date. All the statistical analyses were performed using R version 4.4.0 (R Foundation for Statistical Computing, Vienna, Austria) [22].

## Results

### Institutional data availability

Data completeness and post-implementation follow-up varied substantially across the three participating institutions. Clínica CEDES provided the most robust dataset, with clear pre-implementation information from 2018 to 2021 and post-implementation data from 2022 to 2023. ESE Hospital La Divina Misericordia had pre-implementation data from 2018 to 2023 and only partial post-implementation information from 2024 onwards. ESE Hospital San José de Maicao contributed comprehensive baseline economic data from 2018 to 2023 and post-intervention longitudinal quality indicators from late 2024, although a mature post-implementation economic evaluation was not yet available.

Because of these differences in data maturity and implementation timing, the results are presented separately by institution. These findings should be interpreted as institutional signals associated with the implementation of the multi-component maternal safety and technological strengthening model rather than as isolated effects attributable solely to the obstetric HDU.

### Population, service use, and costs

At Clínica CEDES, the costing dataset included 120,617 patients between July 2018 and September 2023, including 15,327 maternal patients. Annual maternal patient volume increased from 1255 in 2018 to a projected 4370 in 2023; the 2023 estimate was based on partial-year data. Because the economic observation period overlapped with the COVID-19 pandemic, we interpreted changes in service use, referral patterns, resource allocation, and costs during 2020–2021 as contextual temporal variation rather than intervention-related effects.

Annual mean total cost per maternal event differed across institutions and implementation periods (Table 3). At Clínica CEDES, annual mean event costs ranged from COP $1.97 million to COP $2.36 million during the pre-implementation period (2018–2021) and were COP $2.65 million and COP $2.84 million in 2022 and 2023, respectively. Compared with the immediately preceding year, both direct and allocated shared costs increased. These higher early implementation costs likely reflect, at least in part, increased maternal service volume and start-up requirements, including workforce recruitment or reassignment, equipment acquisition or reallocation, and adaptation of the physical area required to operationalise the high-dependency model. Accordingly, early implementation costs should not necessarily be interpreted as steady-state operating costs.

**Table 3:** Annual mean total cost per maternal event (range across years).

INSTITUTION	PERIOD	YEARS	ANNUAL MEAN TOTAL COST PER MATERNAL EVENT*
CEDES	Pre-implementation	2018–2021	COP $1.97–2.36 million
CEDES	Implementation	2022–2023	COP $2.65–2.84 million^a^
Maicao	Pre-implementation baseline	2018–2023	COP $1.24–2.55 million
Divina Misericordia	Pre-implementation	2018–2023	COP $1.93–3.00 million
Divina Misericordia	Early implementation	2024	COP $2.88 million^b^

*Costs were estimated by bottom-up micro-costing as directly billed costs within each ≤45-day maternal episode plus allocated personnel, equipment, infrastructure, and operating costs. Values represent the range of annual mean costs per maternal event within each period.

^a^ Higher early implementation costs at CEDES partly reflected increased maternal service volume and start-up investments, including personnel, equipment, and HDU facility adaptation, and may therefore exceed steady-state operating costs.

^b^ The 2024 Divina Misericordia estimate reflects partial early implementation follow-up and should not be interpreted as a steady-state postimplementation cost.

ESE Hospital San José de Maicao provided care to 11,584 maternal patients between 2018 and 2023. The annual mean total cost per maternal event ranged from COP $1.24 million to COP $2.55 million over this period, peaking in 2023. The higher cost in 2023 was driven mainly by direct accounting costs, while allocated shared costs fluctuated across years. Because the HDU became operational only after the period covered by the available economic data, these estimates represent baseline cost characterisation rather than post-implementation economic effects.

ESE Hospital La Divina Misericordia contributed 165,277 patients, including 27,500 maternal patients, over the available 2018–2024 observation period; 2024 data represented partial follow-up. Maternal service volume was highest in 2021 and 2022, then declined. During the 2018–2023 pre-implementation period, annual mean total cost per maternal event ranged from COP $1.93 million to COP $3.00 million. The partial 2024 implementation estimate was COP $2.88 million per maternal event, slightly below the 2023 value of COP $3.00 million. Given the limited duration of post-implementation follow-up, this early estimate should not be interpreted as the steady-state operating cost of the model.

### Clinical outcomes and exploratory cost-effectiveness

At Clínica CEDES, the post-implementation period was characterised by favourable changes in several SMM indicators. Comparing the selected pre-implementation reference period (2018–2019) with 2022–2023, mean total length of stay decreased from 5.72 to 5.30 days, mean ICU length of stay from 2.48 days to zero, the mean number of SMM complications from 3.57 to 1.70, organ dysfunctions from 0.78 to 0.61, and MM from 2.90% to 0.62%. However, ICU utilisation had already declined to zero by 2021, before formal HDU implementation in January 2022; therefore, changes in ICU use should not be attributed solely to the intervention. Exploratory ICER estimates showed higher costs alongside improved effectiveness for several intermediate morbidity and resource-use outcomes. These findings suggest favourable value signals for selected outcomes under the study’s exploratory decision framework, but the intervention should not be interpreted as cost-saving or as demonstrating definitive overall cost-effectiveness.

At ESE Hospital La Divina Misericordia, the limited 2024 post-implementation period showed mixed clinical signals. Mean hospital length of stay decreased from 3.30 to 1.30 days, and no maternal deaths were recorded among the 54 women included in the early implementation period, compared with 5 deaths among 165 women in the selected historical comparator. No ICU admissions were recorded during the implementation period. Other morbidity indicators were heterogeneous: the mean number of SMM-related diseases decreased slightly, whereas the mean number of complications and organ dysfunctions increased. Exploratory ICER estimates were favourable for selected outcomes, but the short follow-up, small post-implementation sample, and mixed morbidity findings preclude firm conclusions regarding steady-state clinical effectiveness or cost-effectiveness.

At ESE Hospital San José de Maicao, a mature post-implementation economic or conventional pre/post effectiveness comparison was not available because the HDU became operational only after the 2018–2023 economic baseline period. Although longitudinal SIVIGILA data showed favourable changes in several maternal morbidity indicators before implementation, these trends were treated as contextual baseline information and were not attributed to the HDU intervention.

### Longitudinal quality indicators

At Clínica CEDES, monthly quality indicator trends showed a mixed descriptive pattern during the available post-implementation period (Figure 1A–C). The SI showed intermittent peaks and an apparent upward pattern, whereas the WAOS and AOI appeared to decrease over time. However, ITS regression analyses did not demonstrate statistically significant changes in pre-intervention trend, immediate level, or post-implementation slope for any of the total indicators. For WAOS, the pre-intervention trend was *β* = −0.019 (standard error [SE] 0.716; *P* = 0.979), the immediate level change was *β* = 1.355 (SE 4.608; *P* = 0.771), and the change in trend was *β* = −0.038 (SE 0.734; *P* = 0.959). For AOI total, the corresponding estimates were *β* = −0.003 (SE 0.035; *P* = 0.935), *β* = −0.128 (SE 0.234; *P* = 0.586), and *β* = −0.021 (SE 0.036; *P* = 0.569). For SI total, the estimates were *β* = −0.0001 (SE 0.0551; *P* = 0.998), *β* = 0.063 (SE 0.354; *P* = 0.859), and *β* = 0.031 (SE 0.056; *P* = 0.588), respectively.

**Figure 1A–C:**
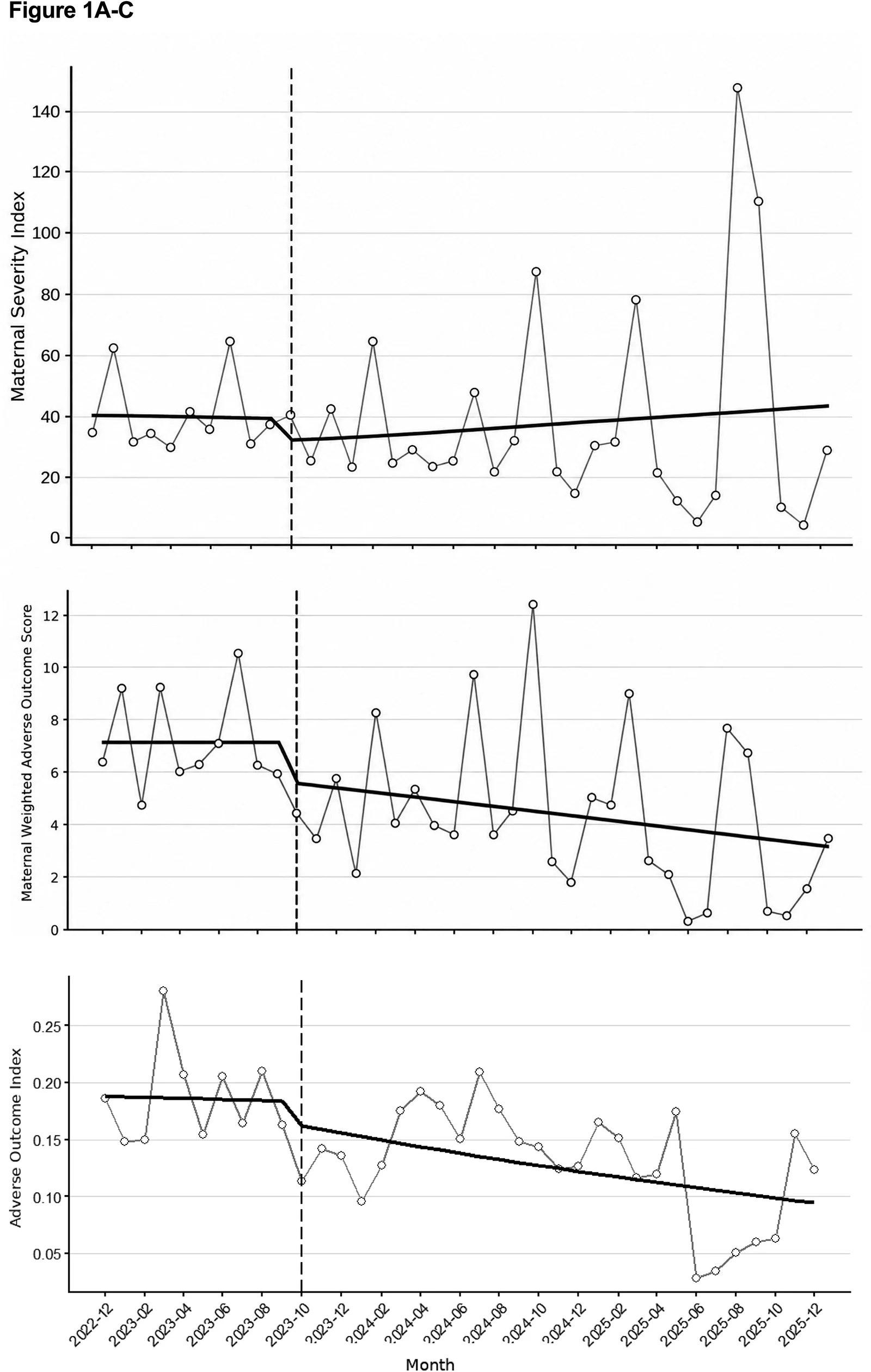
Combined longitudinal indicator panel for Clínica CEDES during the post-implementation monitoring period. Panel A shows the severity index (SI), Panel B the weighted adverse outcome score (WAOS), and Panel C the adverse outcome index (AOI). Open circles connected by grey lines indicate monthly observations, and solid black lines represent fitted temporal trends. Vertical dashed lines mark the monitoring reference point used in each panel; these correspond to the analysed post-implementation indicator window rather than the formal start of the multi-component institutional model, which began in January 2022. Descriptively, WAOS and AOI decreased over time, whereas SI showed intermittent peaks and an apparent upward pattern. Interrupted time series analyses did not identify statistically significant changes in pre-intervention trend, immediate level change, or post-implementation slope for any total indicator: WAOS trend *β* = −0.019 (SE 0.716; *P* = 0.979), level *β* = 1.355 (SE 4.608; *P* = 0.771), slope change *β* = −0.038 (SE 0.734; *P* = 0.959); AOI trend *β* = −0.003 (SE 0.035; *P* = 0.935), level *β* = −0.128 (SE 0.234; *P* = 0.586), slope change *β* = −0.021 (SE 0.036; *P* = 0.569); SI trend *β* = −0.0001 (SE 0.0551; *P* = 0.998), level *β* = 0.063 (SE 0.354; *P* = 0.859), slope change *β* = 0.031 (SE 0.056; *P* = 0.588).

At ESE Hospital San José de Maicao, longitudinal quality indicators provided stronger evidence of improvement following implementation (Figure 2A–C). For WAOS total, the pre-intervention trend was increasing and statistically significant (*β* = 0.784; SE 0.359; *P* = 0.041), followed by a significant immediate reduction in level after implementation (*β* = −8.302; SE 2.283; *P* = 0.002) and a significant decrease in the post-implementation slope (*β* = −1.205; SE 0.396; *P* = 0.006). These findings suggest both an abrupt reduction in the weighted burden of adverse outcomes and a sustained downward trajectory thereafter. For AOI total, there was a significant immediate reduction after implementation (*β* = −1.130 on the logit scale; SE 0.337; *P* = 0.003), indicating a reduction in the frequency of adverse obstetric outcomes, although neither the pre-intervention trend (*β* = −0.010; SE 0.038; *P* = 0.799) nor the post-implementation change in slope (*β* = −0.006; SE 0.048; *P* = 0.901) was statistically significant. For the SI, the pre-intervention trend was significantly increasing (*β* = 0.234; SE 0.104; *P* = 0.036), while the post-implementation change in slope was significantly negative (*β* = −0.268; SE 0.115; *P* = 0.030), suggesting interruption of a previously rising severity trajectory. The immediate level change for SI was not statistically significant (*β* = −0.638; SE 0.662; *P* = 0.347).

**Figure 2A–C:**
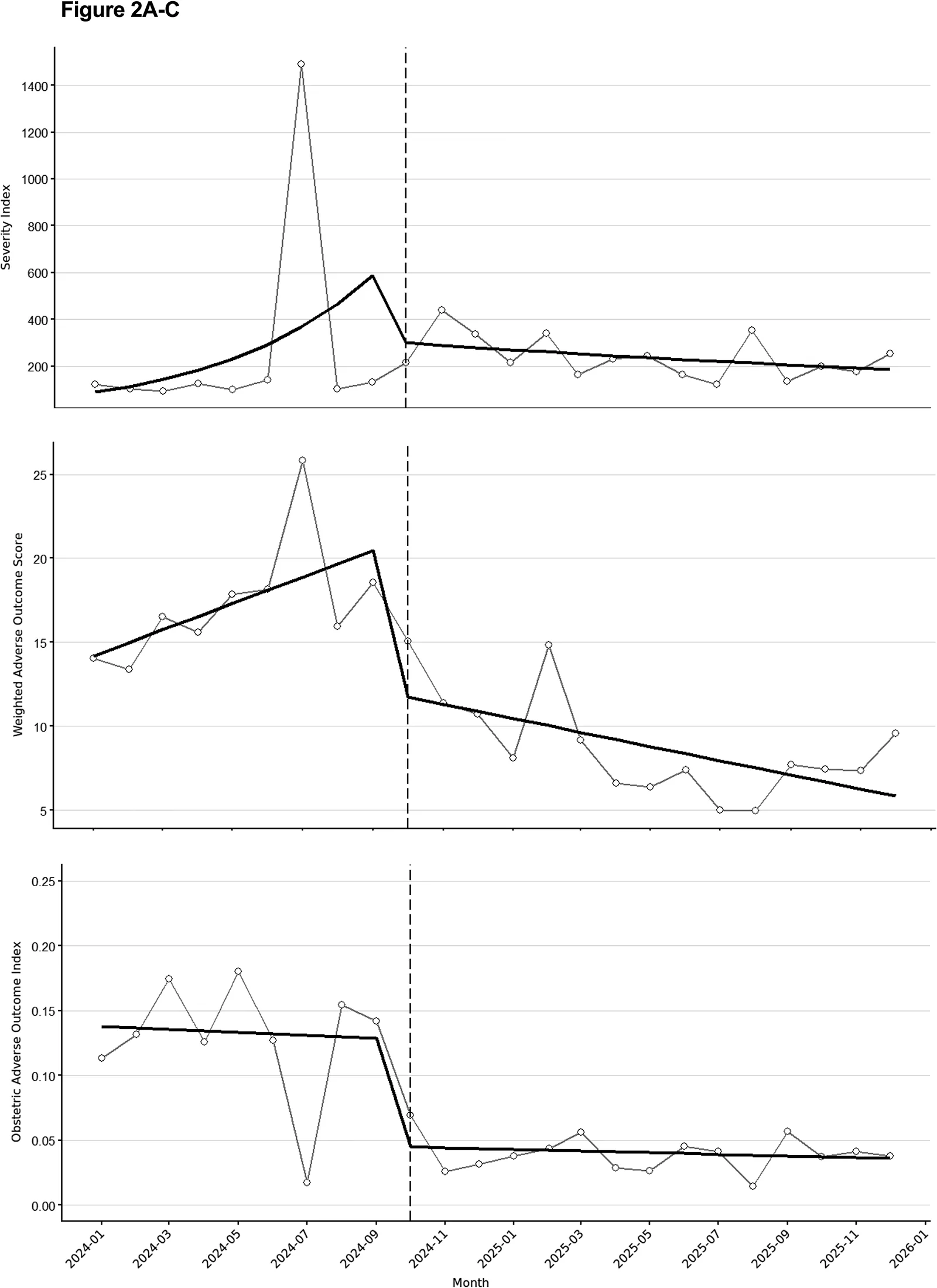
Longitudinal obstetric quality indicators at ESE Hospital San José de Maicao. Panel A shows the severity index (SI), Panel B the weighted adverse outcome score (WAOS), and Panel C the adverse outcome index (AOI), following the same sequence used for Clínica CEDES. Open circles connected by grey lines indicate monthly observations, and solid black lines represent fitted trends before and after implementation. The vertical dashed line marks HDU implementation in October 2024. The results showed a favourable post-implementation pattern: SI had a significantly increasing pre-intervention trend (*β* = 0.234; SE 0.104; *P* = 0.036) followed by a significant negative slope change after implementation (*β* = −0.268; SE 0.115; *P* = 0.030), although the immediate level change was not statistically significant (*β* = −0.638; SE 0.662; *P* = 0.347); WAOS showed a significantly increasing pre-intervention trend (*β* = 0.784; SE 0.359; *P* = 0.041), a significant immediate level reduction (*β* = −8.302; SE 2.283; *P* = 0.002), and a significant decrease in post-implementation slope (*β* = −1.205; SE 0.396; *P* = 0.006); AOI showed a significant immediate post-implementation level reduction (*β* = −1.130 on the logit scale; SE 0.337; *P* = 0.003), whereas the pre-intervention trend (*β* = −0.010; SE 0.038; *P* = 0.799) and post-implementation slope change (*β* = −0.006; SE 0.048; *P* = 0.901) were not statistically significant.

Taken together, these findings indicate that implementation of the multi-component model at Maicao was associated with significant improvements in the frequency and weighted burden of adverse obstetric outcomes and with a favourable change in the trajectory of severity. National government intervention may have facilitated these changes by strengthening governance and operational alignment.

At ESE Hospital La Divina Misericordia in Magangué, the available WAOS series showed an apparently favourable descriptive pattern after implementation, with an overall decrease despite intermittent peaks (Figure 3). However, the quasi-Poisson ITS model did not identify statistically significant changes in the temporal or intervention-related terms. The pre-intervention trend was *β* = −0.274 (SE 0.261), corresponding to a rate ratio of 0.760 (95% confidence interval [CI] 0.439–1.240; *P* = 0.306). The immediate level change after intervention was *β* = 0.193 (SE 0.669), corresponding to a rate ratio of 1.210 (95% CI 0.359–5.220; *P* = 0.776). The change in trend after intervention was *β* = 0.265 (SE 0.262), corresponding to a rate ratio of 1.300 (95% CI 0.795–2.260; *P* = 0.326). Although the intercept was statistically significant (*β* = 2.656; SE 0.593; rate ratio 14.20; 95% CI 4.06–42.70; *P* = 0.0003), this parameter represents the estimated baseline rate and should not be interpreted as an intervention effect. Therefore, although the descriptive trajectory may suggest early improvement, longer follow-up and additional data are needed to determine whether these signals represent a sustained intervention effect. Table 4 summarises an integrated institutional interpretation of the economic, clinical, and longitudinal quality findings.

**Figure 3:**
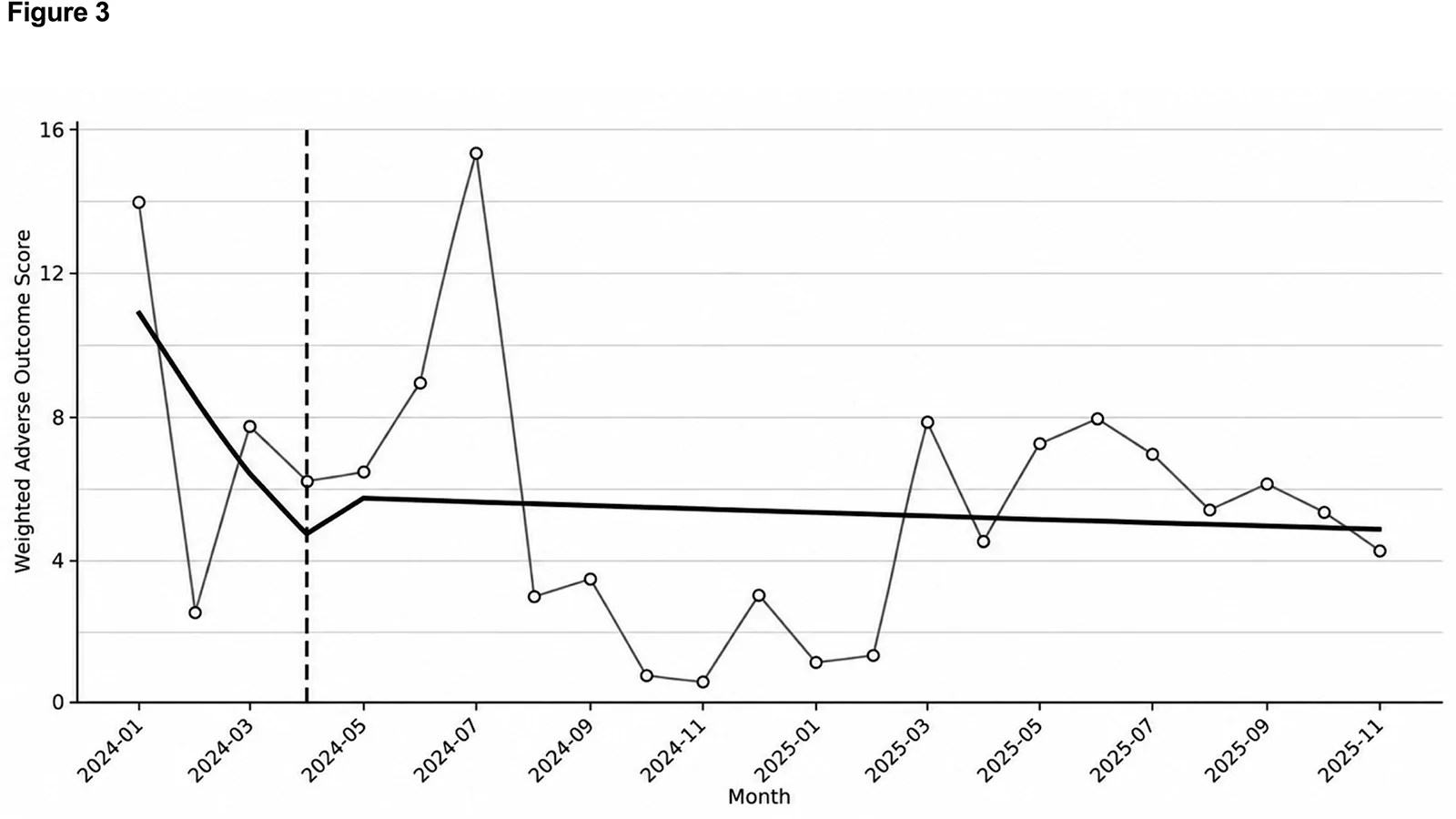
Weighted adverse outcome score at ESE Hospital La Divina Misericordia, Magangué. Monthly trends in the weighted adverse outcome score at ESE Hospital La Divina Misericordia, January 2024–November 2025. Open circles connected by grey lines indicate monthly observations, and the solid black line represents the fitted temporal trend. The vertical dashed line marks HDU implementation in April 2024. The quasi-Poisson interrupted time series model did not identify statistically significant intervention-related changes: pre-intervention trend rate ratio 0.760 (95% CI 0.439–1.240; *P* = 0.306), immediate level-change rate ratio 1.210 (95% CI 0.359–5.220; *P* = 0.776), and post-implementation trend-change rate ratio 1.300 (95% CI 0.795–2.260; *P* = 0.326).

**Table 4:** Integrated institutional interpretation.

INSTITUTION	ECONOMIC PATTERN	CLINICAL PATTERN	QUALITY INDICATOR PATTERN	OVERALL INTERPRETATION
CEDES	Higher post-implementation costs	Reduced morbidity and ICU-related indicators	Descriptive decreases in AOI and WAOS; intermittent SI peaks; no statistically significant ITS changes	Improved conventional clinical indicators with higher implementation costs; longitudinal quality signals are descriptive and hypothesis-generating rather than confirmatory
Maicao	Baseline 2018–2023 cost increase; no mature post-implementation economic comparison	No mature conventional pre/post clinical effectiveness comparison; favourable longitudinal quality observations	Significant reductions in WAOS level and slope; significant immediate AOI reduction; favourable SI slope change	Strongest associative longitudinal quality-improvement signal after implementation, although economic and conventional clinical post-implementation comparisons remain immature
Divina Misericordia	Preliminary comparable costs; partial follow-up	Mixed early clinical signals: shorter hospital stay and no recorded deaths, but increases in some morbidity measures	Descriptive WAOS decrease; no statistically significant ITS intervention effect	Mixed early clinical signals and a descriptive WAOS decrease; evidence remains preliminary and requires longer follow-up before sustained effectiveness can be inferred

## Discussion

This multisite implementation evaluation shows that a multi-component maternal safety and technological strengthening model, including obstetric high-dependency capacity, was associated with heterogeneous changes in maternal care processes, costs, SMM indicators, and longitudinal obstetric quality metrics across three high-risk institutions in the Colombian Caribbean.

Overall, the findings support integrated maternal rescue systems but do not show uniform improvement across sites. The robust longitudinal signal was observed at ESE Hospital San José de Maicao, whereas Clínica CEDES and ESE Hospital La Divina Misericordia showed favourable but more cautious descriptive patterns because statistical evidence was limited or follow-up was incomplete.

Institutional patterns differed substantially across governance and operational models. At Clínica CEDES, a private institution providing medium- and high-complexity obstetric care, implementation coincided with higher costs and favourable conventional clinical indicators, although WAOS, AOI, and SI did not change significantly [20, 23]. At ESE Hospital San José de Maicao, a public state-owned hospital under national government intervention, longitudinal indicators showed the clearest statistical improvement, with significant reductions in WAOS level and slope, an immediate AOI reduction, and a favourable SI trajectory. At ESE Hospital La Divina Misericordia, a public state-owned hospital with private operational support, early clinical and WAOS patterns were mixed and preliminary. These differences suggest that governance, operational alignment, baseline capacity, case-mix, and data maturity shaped implementation value [24].

Key strengths include the real-world evaluation of a multi-component maternal safety and obstetric high-dependency care model in a resource-constrained middle-income region, the inclusion of three distinct organisational models, and the combined assessment of economic, clinical, and longitudinal quality indicators [25].

The study has important limitations. The integrated intervention design prevents attribution to any single component, including HDU capacity. Differences in implementation timing, data maturity, and availability of patient-level severity adjustment limited comparability and precluded pooled estimates. ITS findings should be interpreted as institutional associative signals, especially at CEDES, where monthly variation was substantial, and at Divina Misericordia, where follow-up was short. Pandemic-related variation was treated only as descriptive context and not as evidence of intervention effect.

The economic findings are best interpreted as value signals rather than simple cost changes [26]. At Clínica CEDES, higher post-implementation costs occurred alongside favourable clinical changes, including reductions in intensive care utilisation, SMM-related measures, organ dysfunction, and MM [27]. Start-up investments in training, monitoring, infrastructure, and equipment may raise early costs but still represent good value if they reduce high-cost adverse outcomes, ICU days, complications, or deaths.

Williams et al.’s E-MOTIVE economic evaluation provides a useful benchmark: a calibrated blood-loss drape plus first-response postpartum haemorrhage (PPH) bundle reduced severe PPH and remained cost-effective across country-level analyses, with a small mean incremental cost [28]. Although SAFEX differs in scope and setting, both evaluations support the economic plausibility of maternal safety bundles when implementation costs are accompanied by reductions in severe morbidity, escalation of care, or disability-relevant outcomes.

The longitudinal analyses refine the interpretation of intervention effects. Maicao showed the most consistent pattern, with reduced adverse outcome frequency and weighted burden and improvement in severity trajectory. By contrast, CEDES findings should remain hypothesis-generating, and Divina Misericordia requires longer follow-up before effectiveness can be inferred.

The model’s mechanisms appear interdependent: diagnosis identified gaps, training and protocols improved team response, HDU capacity created an intermediate monitoring platform, and dashboards supported continuous learning. These findings reinforce that obstetric HDUs are most effective when embedded in an integrated maternal rescue system rather than implemented as isolated infrastructure.

For policy and practice, the results suggest that regions with limited intensive care capacity may benefit more from integrated high-dependency care, workforce development, governance alignment, and quality monitoring than from expanding ICU beds alone. Future research should identify which operational practices sustain improvements across private, public, and mixed public–private institutional models.

In conclusion, implementation of a multi-component maternal safety and technological strengthening model that included obstetric high-dependency capacity was associated with heterogeneous but clinically meaningful changes across three Colombian Caribbean institutions. The strongest longitudinal evidence came from Maicao, while CEDES and Divina Misericordia showed favourable but less definitive patterns. These findings suggest that HDU capacity is most likely to improve maternal outcomes when embedded within a broader rescue system combining readiness assessment, trained teams, standardised processes, technological support, governance alignment, and continuous measurement. Longer follow-up, severity-adjusted indicators, and clearer separation of start-up and recurrent costs are needed to assess sustainability, scalability, and value.

## Funders

This study was funded by Ministerio de Ciencia, Tecnología e Innovación (92092).

## Data Availability

Deidentified aggregate data supporting the findings of this study are available from the corresponding author upon reasonable request and subject to institutional, ethical, and data governance approval. Patient-level data cannot be shared publicly because of privacy, confidentiality, and institutional data protection restrictions.
